# Toxicological safety assessment of a water extract of *Lithocarpus litseifolius* by a 90-day repeated oral toxicity study in rats

**DOI:** 10.3389/fphar.2024.1385550

**Published:** 2024-06-20

**Authors:** Jinfeng Ma, Yujia Wei, Jingfeng Sun, Fang Tan, Penghe Liu, Guangqiu Qin

**Affiliations:** Department of Preventive Medicine, Guangxi University of Chinese Medicine, Nanning, China

**Keywords:** *Lithocarpus litseifolius*, Sweet Tea, subchronic toxicity, safety assessment, oral exposure

## Abstract

*Lithocarpus litseifolius* although known as “Sweet Tea” (ST), has been traditionally accepted as a daily beverage and used as a folk medicine in southern China with little understanding of its potential toxicity. This study evaluated the safety of a water extract of ST by a subchronic toxicity study in Sprague-Dawley rats. A total of 80 rats were randomized divided into 4 groups with 10 males and 10 females in each group, treated with 2000, 1,000, 500 and 0 mg/kg body weight of ST extract by gavage for 90 days, respectively. The results of the study showed that ST extract did not induce treatment-related changes in the body and organ weight, food intake, blood hematology and serum biochemistry, urine indices, and histopathology in rats. The NOAEL of ST extract was observed to be 2000 mg/kg/day for rats of both sexes. These results indicated that ST extract was of low toxicity in the experimental conditions of the current study and had the potential for application in food-related products.

## 1 Introduction

*Lithocarpus litseifolius* (Hance) Chun, an evergreen tree belongs to the family Fagaceae, has about 40 alternative names, with the most commonly used being “Sweet Tea” (ST). This name first appeared in the Song Dynasty’s “Annals of Qingshuiyan in Anxi” (Chen, 1996). Wild ST grows primarily in the southern region of the Yangtze River in China, including Jiangxi, Guangxi and Hunan provinces. Traditionally, tender leaves and roots of ST are used to brew tea or stew soup for consumption. The use of tender leaves of ST as tea can be traced back to 423 AD (Chongqing Beibei District Chronicle Compilation Committee, 1989). For centuries, its roots, stems, and leaves have been widely used as a traditional herb in southern China to treat hypertension, obesity and hyperlipidemia (Wu et al., 2019).

Chemical analysis revealed that leaves of ST are rich of flavonoids (including phloridzin and trilobatin) and polyphenolic compounds (Li et al., 2014). The results of modern biomedical studies have shown that the chemical components of ST have multiple biological activities. For example, the extract of ST was observed to have broad-spectrum antibacterial activity toward Gram-positive bacteria and fungi (Wang et al., 2020). Different fractions from ST leaves, including total extract, petroleum ether fraction, n-butyl-alcohol fraction, water fraction, phlorizin, phloretin and 2′-O-acetylph loridzin could significantly promote the glucose consumption of insulin-resistant HepG2 cells and improve the insulin resistance of HepG2 cells (Pan et al., 2015). Tea extract mainly composed of ST was observed to reduce uric acid in mice with hyperuricemia nephropathy, possibly through the inhibition of uric acid reabsorption (Chen et al., 2024). Trilobatin, phlorizin, isoquercitrin and other components of ST could improve ulcerative colitis in model mice, mainly through the regulation of PI3K-AKT and TNF signaling pathways that are related to inflammation, immunity, anti-oxidation, and the intestinal barrier (Liu, 2023). Historical use and emerging preclinical and clinical evidence suggest that there is potential for the extract of ST to be used in teas or as a dietary supplement, and it has been applied in health food-related products (Ji et al., 2020; Huang, 2021; Chen et al., 2023).

Although the biological effects of ST and its extract have been widely recognized and confirmed by modern biomedical research, there is still relatively little understanding of its potential toxicity. There are no records of dietary taboos or toxicity of ST in traditional Chinese medicine works and literature throughout history (Lin et al., 2023). A previous study reported that 25-weeks repeated oral administration of ST extract at dose of 2.0 g/kg led to reversible damage to the liver function of Wistar rats (Zeng et al., 2010). Another study reported that three phlorizin derivatives and four dihydrochalcones isolated from the leaves of ST were shown to be non-cytotoxic when tested against A549, HeLa, HepG2, and MCF-7 cell lines (Wei et al., 2020). The objective of the present study was to evaluate the subchronic toxicity of a water-extract of ST in rats to provide necessary information for safety assessment of ST in food-related products.

## 2 Materials and methods

### 2.1 Preparation and standardization of plant extract

The fresh leaves of ST were collected from Bama Yao Autonomous County, Guangxi province, China in the summer of 2018. Leaves were dried in a 60°C oven, powdered and ultrasonically extracted twice with distilled water (1:10, w:v) at 60°C for 1 h each time. The aqueous extract was mixed, filtered, concentrated using a rotary evaporator (Heidolph Advantage ML/G3, Germany), and freeze-dried to obtain a powdered extract (1 g of powder is equivalent to 31.8 g of dried leaves). The phloridzin content of the extract was determined to be 84.3 mg/g using high performance liquid chromatography, following the method reported (Chen et al., 2017). The extract was stored at −20 °C and diluted with distilled water before being used.

### 2.2 Experimental animals

Three-week-old specific pathogen-free Sprague-Dawley (SD) rats were purchased from the Medical Experimental Animal Center of Guangdong Province (Guangzhou, China). Animals were housed in an environment with a 12 h light/dark cycle, a temperature of 23°C ± 1°C and a relative humidity of 60% ± 5%. Animals were housed in polycarbonate cages with unrestricted access to standard diets and distilled water. They were acclimated for 3 days before the experiments. The protocol of this study was reviewed and approved by the Animal Experimentation Ethics Committee of Guangxi University of Chinese Medicine.

### 2.3 Animal grouping and exposure

A 90-day subchronic oral toxicity study was conducted following a standard protocol established by the National Health Commission of China (National Health Commission of China, 2015; Qin et al., 2020). A total of 80 healthy male and female SD rats were randomly assigned to 3 treatment groups and 1 control group, with 10 males and 10 females in each group. Animals in the treatment groups were administered 2000, 1,000, and 500 mg/kg body weight of ST extract (dissolved in distilled water) by gavage once per day in the morning for 90 days, respectively. In contrast, those in the control group were given 10 mL/kg of distilled water daily by gavage. Doses were determined based on our preliminary, unpublished acute toxicity study on this ST extract, which concluded with a NOAEL of >5,000 mg/kg body weight in Kunming mice. Animals of different sexes were housed separately in polycarbonate cages, with a maximum of three animals per cage. Conventional diets and water were freely available to all animals during the experiment.

### 2.4 Clinical observation

Clinical signs and behavioral symptoms were recorded daily, including hair condition, skin appearance, eyes health, mucous membranes status, secretions, excretions, respiratory system function, nervous system responses and behavioral manifestations. Individual body weight was recorded weekly. Food consumption was recorded twice a week throughout the study. Feed efficiency was calculated as follows: Feed efficiency (%) = body weight gain (g)/food intake (g) × 100% (Qin et al., 2020).

### 2.5 Ophthalmological examination

The ophthalmological examination was conducted during the acclimation period and repeated on the final day of the exposure. The cornea, lens, bulbar conjunctiva, and iris were observed using an ophthalmoscope.

### 2.6 Hematological and serum biochemical analyses

After 90 days of exposure, rats were fasted overnight. Blood samples were collected from the arteria abdominalis under pentobarbital anesthesia. Hematological examination of blood was conducted using a Sysmex XT-1800 automated hematological analyzer (Sysmex, Kobe, Japan). The hematological indexes examined included the following: white blood cell count, red blood cell count, hemoglobin concentration, hematocrit, platelet count, mean platelet volume, mean corpuscular volume, mean corpuscular hemoglobin, mean corpuscular hemoglobin concentration, number and percentage of neutrophils, number and percentage of lymphocytes, number and percentage of monocytes, number and percentage of eosinophils, number and percentage of basophils.

Serum from rats was collected by centrifuging approximately 4 mL of whole blood at 2,500 × rpm for 10 min. The activity of aspartate transaminase and alanine transaminase in serum was analyzed using commercial kits (Beijing Wantai BioPharm, Beijing, China). Biochemical indexes of serum, including blood urea nitrogen, creatinine, total cholesterol, triglycerides, total protein, albumin, and glucose, were analyzed using an Olympus AU400 analyzer (Olympus, Tokyo, Japan).

### 2.7 Urinalysis

Urine samples were collected from the bladders of rats after blood collection using syringes. Urine indicators, including specific gravity, pH, white blood cells, ketone bodies, nitrite, urobilinogen, bilirubin, protein, glucose, occult blood, creatinine, calcium, and microalbumin, were analyzed using a Urit-500B urine chemistry analyzer (Urit, Guilin, China).

### 2.8 Necropsy

After being sacrificed by exsanguination from the abdominal aorta, a visual pathological examination was conducted on all rats. The weights of the liver, spleen, kidneys, testes, ovaries, brain, heart, thymus, adrenal glands, epididymis and uterus were measured. Relative organ weight was determined as organ weight divided by body weight and then multiplied by 100%.

### 2.9 Histopathological examination

Samples of organs and tissues were preserved for histopathological examination, including the brain, thyroid gland, liver, spleen, pancrea, heart, kidneys, adrenal gland, stomach, mesenteric lymph nodes, small intestine, jejunum, ileum, prostate, bladder, testes, and ovaries. Samples were fixed in 4% neutral buffered formaldehyde, embedded in paraffin, and stained with Giemsa. Pathological sections of organs and tissues were examined under a Leica DM 6000B optical microscope (Wetzler, Germany). The number of animals with histopathological lesions was recorded. The types of histopathological lesions were recorded, and the degree of each lesion was scored into four levels: normal (0), mild (1), moderate (2), and severe (3).

### 2.10 Statistical analysis

The data were analyzed using SPSS v16.0 (SPSS Inc., Chicago, United States). The homogeneity of variances in the data was assessed using Bartlett’s test. The data from the treatment groups were compared to those of the control group using one-way ANOVA followed by Dunnett’s test. A *p*-value of ≤0.05 was considered statistically significant.

## 3 Results

### 3.1 Clinical and ophthalmological examination

During the 90-day toxicity study, no mortality occurred, and there were no treatment-related clinical changes or abnormal behavior observed. No alterations were observed on ophthalmologic examinations before and on Day 90 of the treatment.

### 3.2 Body and organ weights

**FIGURE 1:**
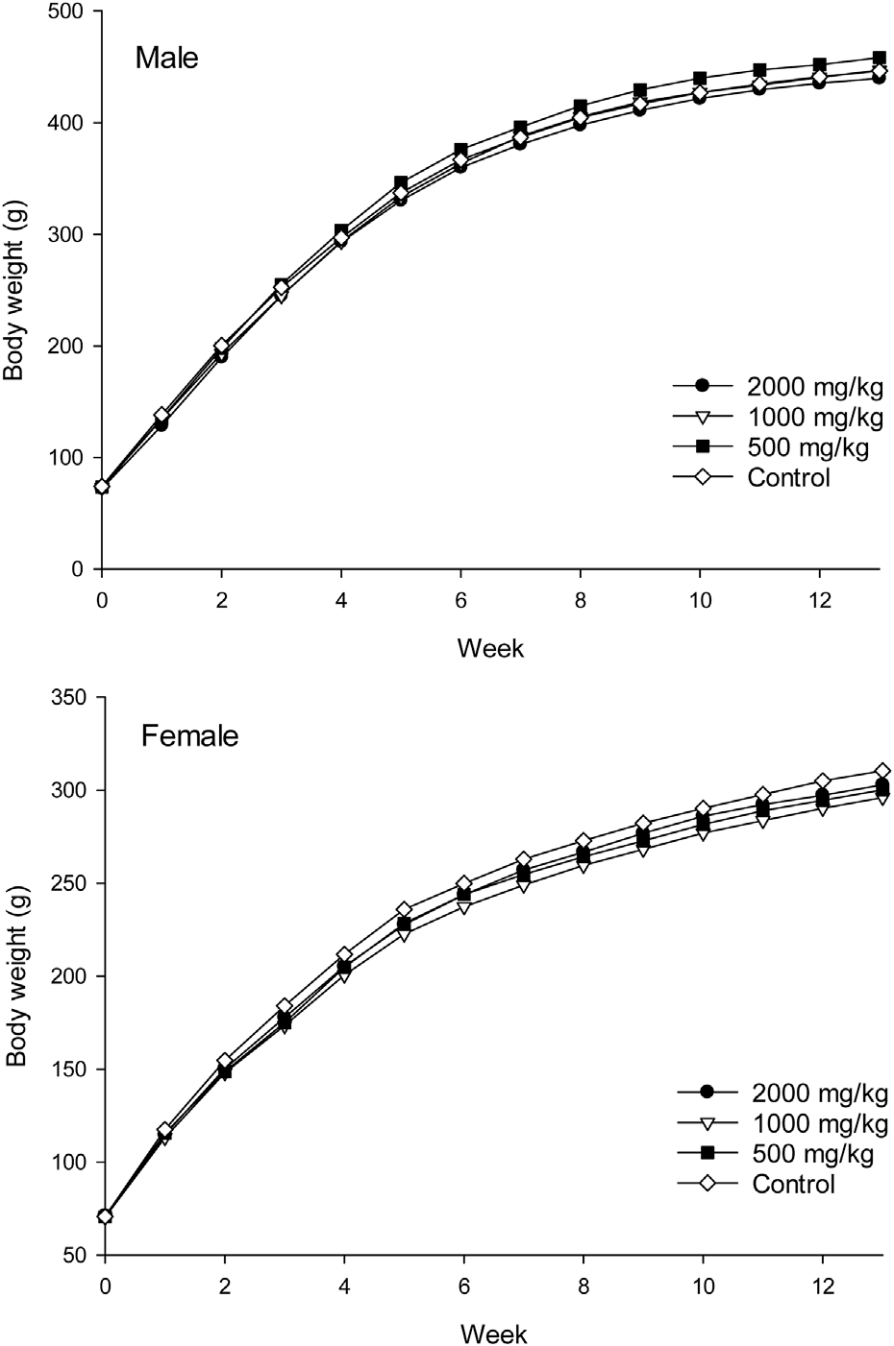
Average body weights of rats treated with ST extract for 90 days (represent by mean, n = 10).

Body weights and weight gains of the treated groups were comparable to those of the control group for both sexes (*p* > 0.05, Figure 1; Table 1). No significant difference was observed in the total food intake and average feed efficiency of rats between the treated and control groups of both sexes (*p* > 0.05, Table 1). The absolute weights and relative weights of major organs in the treated groups of rats were similar to those of the control group for both sexes (*p* > 0.05, Tables 2, 3).

**TABLE 1:** Body weight gain, food intake and food utilization rate of rats treated with ST extract for 90 days.

Sex	Dose (mg/kg)	Total body weight gain (g)			Total food intake (g)			Average feed efficiency (%)		
Male	2,000	367.4	±	26.0	2,463.7	±	60.5	14.9	±	1.1
	1,000	374.0	±	24.4	2,432.4	±	116.7	15.4	±	0.8
	500	384.8	±	28.5	2,532.9	±	116.8	15.2	±	0.7
	Control	372.4	±	29.2	2,447.6	±	93.9	15.2	±	0.8
Female	2,000	231.8	±	13.0	2,112.0	±	143.7	11.0	±	0.7
	1,000	225.7	±	13.5	2054.4	±	157.3	11.0	±	0.9
	500	229.6	±	18.9	2044.1	±	150.3	11.3	±	0.9
	Control	239.7	±	15.5	2,131.3	±	182.2	11.3	±	0.6

Note: values represent mean ± standard deviation of 10 rats. Feed efficiency (%) = (total body weight gain/total food intake) ×100%. Values of the treatment groups did not differ statistically from the control according to one-way ANOVA, at *p* < 0.05.

**TABLE 2:** Absolute organ weights of rats treated with ST extract for 90 days.

Dose (mg/kg)	Liver			Kidneys			Spleen			Testes/ovaries			Brain			Heart			Thymus			Adrenal			Epididymis/uterus		
Male																											
2,000	10.11	±	0.57	2.88	±	0.25	0.70	±	0.04	3.11	±	0.15	1.596	±	0.104	1.249	±	0.134	0.407	±	0.081	0.064	±	0.007	1.125	±	0.146
1,000	10.45	±	0.60	2.83	±	0.22	0.73	±	0.08	3.11	±	0.20	1.747	±	0.201	1.207	±	0.145	0.456	±	0.102	0.066	±	0.003	1.094	±	0.138
500	10.64	±	0.77	2.98	±	0.22	0.75	±	0.07	3.14	±	0.34	1.670	±	0.183	1.289	±	0.139	0.414	±	0.068	0.068	±	0.010	1.026	±	0.160
Control	10.27	±	0.78	2.86	±	0.25	0.75	±	0.08	3.02	±	0.22	1.669	±	0.160	1.291	±	0.088	0.431	±	0.111	0.065	±	0.009	1.071	±	0.153
Female																											
2,000	7.56	±	0.36	1.99	±	0.11	0.60	±	0.10	0.158	±	0.016	1.905	±	0.183	0.963	±	0.069	0.447	±	0.018	0.078	±	0.009	0.603	±	0.073
1,000	7.40	±	0.47	1.91	±	0.15	0.58	±	0.08	0.148	±	0.012	1.880	±	0.203	1.014	±	0.116	0.448	±	0.024	0.068	±	0.011	0.613	±	0.063
500	7.46	±	0.58	1.91	±	0.14	0.57	±	0.10	0.153	±	0.016	1.860	±	0.162	1.036	±	0.108	0.448	±	0.034	0.069	±	0.008	0.590	±	0.076
Control	7.78	±	0.40	1.97	±	0.14	0.62	±	0.07	0.158	±	0.011	1.953	±	0.198	1.070	±	0.089	0.458	±	0.025	0.071	±	0.008	0.634	±	0.060

Note: values represent mean ± standard deviation of 10 rats. Values of the treatment groups did not differ statistically from the control according to one-way ANOVA, at *p* < 0.05.

**TABLE 3:** Relative organ weights of rats treated with ST extract for 90 days.

Dose (mg/kg)	Liver			Kidneys			Spleen			Testes/ovaries			Brain			Heart			Thymus			Adrenal			Epididymis/uterus		
Male																											
2,000	2.399	±	0.078	0.683	±	0.035	0.167	±	0.009	0.738	±	0.029	0.380	±	0.031	0.296	±	0.027	0.096	±	0.017	0.015	±	0.001	0.267	±	0.037
1,000	2.441	±	0.035	0.661	±	0.040	0.169	±	0.013	0.726	±	0.032	0.401	±	0.036	0.281	±	0.026	0.106	±	0.019	0.015	±	0.002	0.256	±	0.039
500	2.420	±	0.061	0.679	±	0.038	0.170	±	0.010	0.712	±	0.038	0.380	±	0.031	0.294	±	0.032	0.095	±	0.016	0.015	±	0.002	0.234	±	0.040
Control	2.401	±	0.054	0.669	±	0.039	0.175	±	0.010	0.707	±	0.028	0.390	±	0.034	0.303	±	0.021	0.101	±	0.024	0.015	±	0.002	0.250	±	0.034
Female																											
2,000	2.603	±	0.032	0.684	±	0.017	0.205	±	0.034	0.055	±	0.005	0.655	±	0.047	0.332	±	0.029	0.154	±	0.005	0.027	±	0.002	0.208	±	0.025
1,000	2.604	±	0.032	0.674	±	0.030	0.206	±	0.029	0.052	±	0.003	0.662	±	0.054	0.357	±	0.036	0.158	±	0.004	0.024	±	0.003	0.216	±	0.025
500	2.592	±	0.030	0.664	±	0.039	0.197	±	0.032	0.053	±	0.004	0.647	±	0.040	0.361	±	0.032	0.156	±	0.004	0.024	±	0.003	0.204	±	0.019
Control	2.616	±	0.019	0.662	±	0.028	0.208	±	0.026	0.053	±	0.004	0.656	±	0.050	0.360	±	0.033	0.154	±	0.004	0.024	±	0.002	0.214	±	0.022

Note: values represent mean ± standard deviation of 10 rats. Relative organ weight (%) = (absolute organ weight/fasting body weight) ×100%. Values of the treatment groups did not differ statistically from the control according to one-way ANOVA, at *p* < 0.05.

### 3.3 Blood hematology and serum biochemistry

For blood hematology, in male rats at a dose of 2000 mg/kg, the hemoglobin (HGB) level was significantly higher than in the control group (*p* < 0.05, Table 4). The red blood cell (RBC) levels increased significantly in female rats treated with 500 mg/kg ST extract compared to those in the control group. Platelet (PLT) and white blood cell (WBC) count, as well as their ratios, were not affected by the extract treatment.

**TABLE 4:** Hematological indexes of rats treated with ST extract for 90 days.

Sex	Dose (mg/kg)	HGB (g/L)			RBC (10^12^/L)			PLT (10^9^/L)		
Male	2,000	160.8	±	10.9*	7.71	±	0.95	904.9	±	105.7
	1,000	147.9	±	9.4	7.89	±	0.63	871.5	±	151.7
	500	152.4	±	12.5	7.60	±	0.79	809.2	±	137.1
	Control	147.4	±	7.2	7.94	±	1.07	859.3	±	165.9
Female	2,000	144.6	±	8.4	7.72	±	0.45	832.6	±	147.1
	1,000	146.5	±	15.4	8.10	±	0.61	881.3	±	122.6
	500	154.0	±	11.1	8.38	±	0.80*	848.1	±	139.5
	Control	146.7	±	10.1	7.48	±	0.67	880.1	±	96.8

Sex	Dose (mg/kg)	WBC (10^9^/L)			LYM (%)			NEUT (%)			MONO (%)			EO (%)			BASO (%)		
Male	2,000	7.61	±	0.74	80.9	±	3.7	12.4	±	3.5	5.67	±	0.79	0.87	±	0.41	0.21	±	0.14
	1,000	7.39	±	0.71	81.0	±	3.1	12.3	±	3.7	5.65	±	0.89	0.83	±	0.52	0.24	±	0.07
	500	7.66	±	0.99	79.0	±	2.3	14.3	±	2.0	5.73	±	0.99	0.83	±	0.19	0.25	±	0.08
	Control	6.94	±	0.86	82.2	±	4.0	11.4	±	3.8	5.64	±	0.59	0.69	±	0.29	0.15	±	0.10
Female	2,000	7.16	±	0.66	81.6	±	4.5	11.9	±	4.6	5.34	±	1.15	0.97	±	0.33	0.18	±	0.12
	1,000	7.16	±	0.98	80.0	±	2.9	13.8	±	2.7	5.33	±	0.96	0.74	±	0.30	0.21	±	0.06
	500	7.50	±	0.52	82.6	±	3.9	11.0	±	4.2	5.55	±	0.75	0.65	±	0.38	0.20	±	0.08
	Control	7.05	±	1.19	80.2	±	3.1	12.8	±	3.6	5.86	±	0.83	0.93	±	0.35	0.23	±	0.07

Note: values represent mean ± standard deviation of 10 rats. HGB, hemoglobin concentration; RBC, red blood cell count; PLT, platelet count; WBC, white blood cell count; LYM, percent of lymphocytes; NEUT, percent of neutrophils; MONO, percent of monocytes; EO, percent of eosinophils; BASO, percent of basophils. *p < 0.05 compared with the control.

Coagulation indexes, including fibrinogen (FIB), thrombin time (TT), prothrombin time (PT) and activated partial thromboplastin time (APTT) were not affected by the extract treatment and were similar in all groups (Table 5).

**TABLE 5:** Coagulation indexes of rats treated with ST extract for 90 days.

Sex	Dose (mg/kg)	FIB (g/L)			TT (s)			PT (s)			APTT (s)		
Male	2,000	2.47	±	0.24	58.1	±	5.6	11.0	±	0.8	16.0	±	0.7
	1,000	2.38	±	0.18	58.3	±	5.3	11.2	±	0.8	16.2	±	0.8
	500	2.57	±	0.27	58.7	±	3.7	11.2	±	0.9	16.0	±	0.6
	Control	2.50	±	0.23	59.9	±	4.9	10.9	±	0.7	16.0	±	0.8
Female	2,000	2.08	±	0.14	55.7	±	3.8	9.1	±	0.2	16.0	±	0.5
	1,000	1.95	±	0.13	55.5	±	3.3	9.3	±	0.2	15.8	±	0.5
	500	2.03	±	0.14	55.0	±	4.1	9.4	±	0.2	16.2	±	0.5
	Control	2.03	±	0.13	55.3	±	4.4	9.2	±	0.3	15.8	±	0.5

Note: values represent mean ± standard deviation of 10 rats. FIB, fibrinogen; TT, thrombin time; PT, prothrombin time; APTT, activated partial thromboplastin time. Values of the treatment groups did not differ statistically from the control according to one-way ANOVA, at *p* < 0.05.

For serum biochemical indexes, in male rats at 2000 mg/kg, the albumin (ALB) level was slightly but significantly higher than that of the control (*p* < 0.05, Table 6). In female animals, the blood urea nitrogen (BUN) level at 1,000 mg/kg decreased significantly; the chlorine level at 2000 mg/kg was significantly higher than that of the control, while the natrium level at 1,000 mg/kg was significantly lower than that of the control (*p* < 0.05, Table 6).

**TABLE 6:** Serum biochemical indexes of rats treated ST with extract for 90 days.

Sex	Dose (mg/kg)	AST (U/L)			ALT (U/L)			BUN (mmol/L)			CR (μmol/L)			TC (mmol/L)			TG (mmol/L)			TP (g/L)		
Male	2,000	113.8	±	9.4	49.80	±	4.26	6.26	±	0.62	49.44	±	3.98	1.93	±	0.20	1.02	±	0.13	72.32	±	5.60
	1,000	113.4	±	7.9	50.67	±	7.35	5.99	±	0.42	45.85	±	5.61	1.88	±	0.17	1.07	±	0.20	69.59	±	4.75
	500	116.5	±	8.5	52.66	±	7.55	6.05	±	0.42	47.53	±	4.75	1.96	±	0.07	0.96	±	0.19	72.09	±	7.12
	Control	111.5	±	8.9	49.12	±	8.66	6.20	±	0.61	47.62	±	4.27	2.00	±	0.21	1.02	±	0.24	72.55	±	5.37
Female	2,000	115.1	±	6.1	47.73	±	7.97	5.71	±	0.49	48.24	±	2.88	1.91	±	0.23	1.00	±	0.15	73.55	±	4.17
	1,000	119.4	±	6.8	51.20	±	8.28	5.70	±	0.45*	46.39	±	3.80	1.95	±	0.12	0.95	±	0.23	75.64	±	5.21
	500	117.4	±	10.3	48.34	±	6.69	6.09	±	0.45	46.67	±	5.33	1.88	±	0.29	0.96	±	0.26	71.33	±	4.63
	Control	113.1	±	10.5	47.66	±	6.91	6.26	±	0.61	47.06	±	6.58	1.96	±	0.25	0.93	±	0.21	73.17	±	5.27

Sex	Dose (mg/kg)	ALB (g/L)			GLU (mmol/L)			GGT (U/L)			ALP (U/L)			Cl (mmol/L)			K (mmol/L)			Na (mmol/L)		
Male	2,000	36.27	±	2.04*	5.49	±	0.35	0.47	±	0.19	159.0	±	16.1	91.3	±	3.7	7.46	±	0.34	158.1	±	1.6
	1,000	34.41	±	1.20	5.67	±	0.31	0.44	±	0.20	160.3	±	12.2	90.0	±	3.5	7.33	±	0.35	159.8	±	3.4
	500	35.47	±	1.61	5.53	±	0.34	0.52	±	0.18	157.3	±	14.0	93.0	±	2.3	7.43	±	0.35	159.8	±	3.3
	Control	33.66	±	3.05	5.42	±	0.48	0.58	±	0.16	168.1	±	11.7	92.0	±	3.3	7.29	±	0.35	159.6	±	3.1
Female	2,000	35.47	±	1.52	5.46	±	0.40	0.52	±	0.17	145.2	±	13.1	90.8	±	1.4*	7.07	±	0.23	154.7	±	2.2
	1,000	34.65	±	2.18	5.30	±	0.42	0.43	±	0.13	144.6	±	14.5	89.9	±	1.6	7.25	±	0.28	152.9	±	1.6*
	500	35.03	±	2.25	5.29	±	0.34	0.48	±	0.15	148.9	±	18.1	89.5	±	1.7	7.09	±	0.20	156.3	±	2.4
	Control	35.10	±	1.87	5.33	±	0.33	0.48	±	0.19	146.1	±	14.0	88.9	±	1.7	7.11	±	0.29	155.7	±	2.0

Note: values represent mean ± standard deviation of 10 rats. AST, aspartate transaminase; ALT, alanine transaminase; BUN, blood urea nitrogen; CR, creatinine; TC, total cholesterol; TG, triglyceride; TP, total protein; ALB, albumin; GLU, glucose; GGT, glutamyltransferase; ALP, alkaline phosphatase; Cl, chlorine; K, potassium; Na, natrium. *p < 0.05 compared with the control.

### 3.4 Urinalysis

For urinalysis, the urine color and clarity, specific gravity, and pH of the treatment groups were comparable to those of the control group (*p* > 0.05, Table 7). Several elevated values of white blood cells (WBC), protein (PRO), blood (BLD), creatinine (Cr), calcium (Ca), and microalbumin (MA) were observed in all groups (Table 7).

**TABLE 7:** Urinalysis of rats treated with ST extract for 90 days.

Sex	Dose (mg/kg)	Number of abnormal color/clarity	SG			pH			Number of positive										
									WBC	KET	NIT	URO	BIL	PRO	GLU	BLD	Cr	Ca	MA
Male	2,000	0	1.024	±	0.004	7.4	±	0.8	1	0	0	0	0	2	0	1	0	1	2
	1,000	0	1.020	±	0.006	7.3	±	0.9	0	0	0	0	0	3	0	2	0	1	1
	500	0	1.023	±	0.005	7.2	±	0.7	1	0	0	0	0	2	0	2	0	2	2
	Control	0	1.025	±	0.006	7.4	±	0.7	1	0	0	0	0	2	0	1	1	2	2
Female	2,000	0	1.022	±	0.007	7.3	±	0.7	1	0	0	0	0	2	0	2	0	2	1
	1,000	0	1.020	±	0.006	7.2	±	0.6	0	0	0	0	0	1	0	1	0	2	2
	500	0	1.016	±	0.006	7.0	±	0.5	0	0	0	0	0	1	0	3	1	1	1
	Control	0	1.020	±	0.006	7.1	±	0.7	1	0	0	0	0	2	0	2	0	1	1

Note: values represent mean ± standard deviation of 10 rats. SG, specific gravity; WBC, white blood cell; KET, ketone body; NIT, nitrite; URO, urobilinogen; BIL, bilirubin; PRO, protein; GLU, glucose; BLD, occult blood; Cr, creatinine; Ca, Calcium; MA, micro albumin. Values of the treatment groups did not differ statistically from the control according to one-way ANOVA, at *p* < 0.05.

### 3.5 Histopathological examinations

**FIGURE 2:**
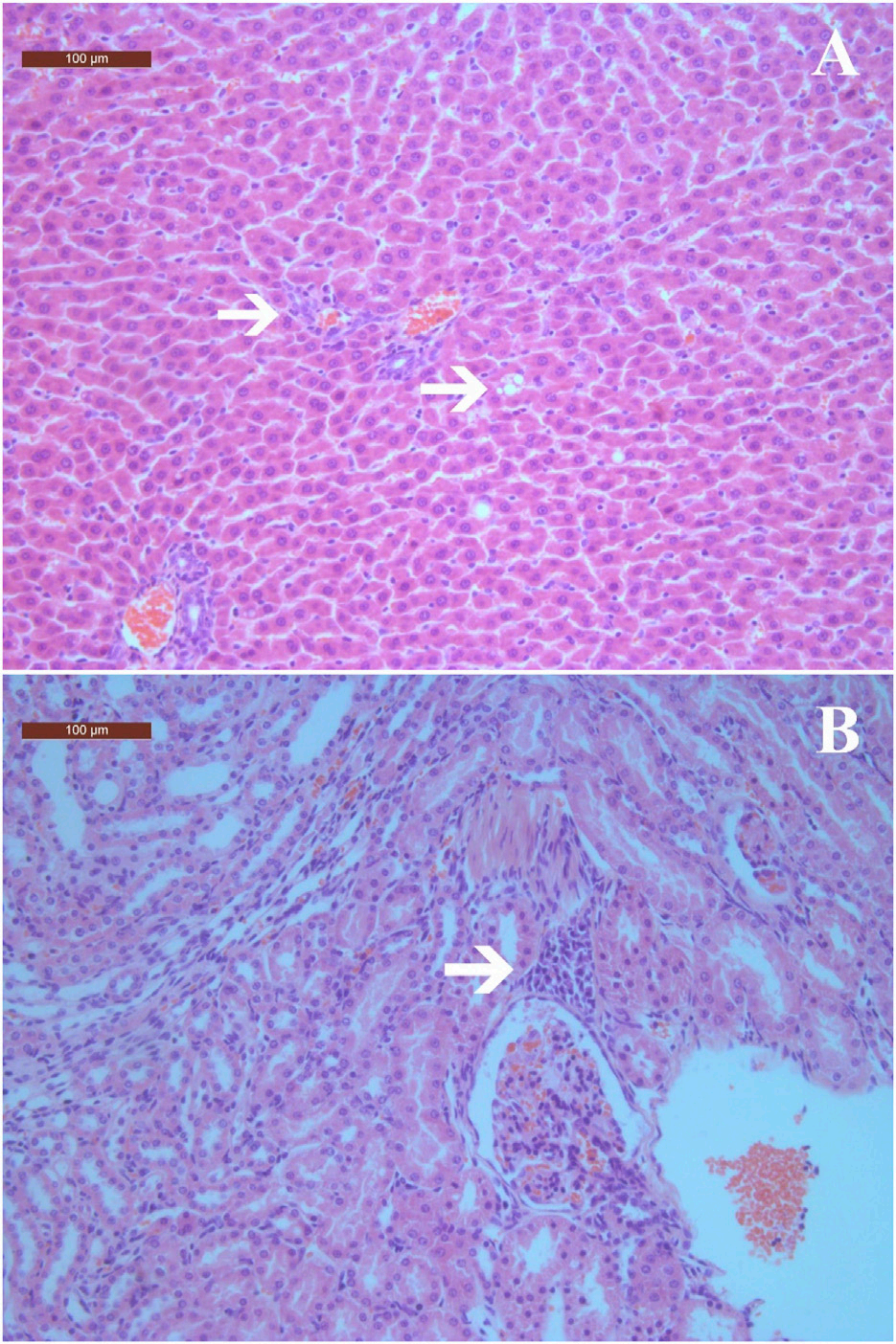
Histopathological changes in liver and kidneys of rats treated with ST extract for 90 days (×100). **(A)** Inflammatory cell infiltration in portal tract areas and fatty degeneration of hepatocytes; **(B)** Cells infiltration in renal interstitium. Arrows indicate histopathological changes. The scale bars are 100 μm.

In the visual pathological examination, no apparent symptoms of pathological lesions were observed in rats from the treatment and control groups. Therefore, histopathological examinations were conducted only in the highest dose (2000 mg/kg) group and the control group. In the histopathological examinations, mild histopathological changes, including inflammatory cell infiltration and fatty degeneration of hepatocytes, as well as cell infiltration in the renal cortex of kidneys were observed in both the treatment and control groups (Figure 2 and Table 8). Scoring of histopathological lesions showed no statistical difference between the treatment and control groups for both sexes (*p* > 0.05, data not shown).

**TABLE 8:** Histopathology examination of rats treated with ST extract for 90 days.

Organs	Histopathological changes	Male		Female	
		2000 mg/kg	Control	2000 mg/kg	Control
Liver	Inflammatory cell infiltration in portal duct areas	3	2	2	1
	Mild fatty degeneration of hepatocytes	1	2	1	2
Kidneys	Cell infiltration in renal cortex	2	2	2	1

Note: values represent numbers of rats with histopathological changes in 10 rats of each group.

## 4 Discussion

The extract of ST leaves has been shown to have various biological effects, including hypoglycemic (Liu et al., 2020; Li et al., 2022), hypolipidemic, antioxidant, and antimicrobial effects (Wu et al., 2019; Wang et al., 2020; Fu, 2023), and was developed to health food and traditional Chinese medicine (Liu, 2017; Wu et al., 2019; Huang, 2021). In 2017, ST leaf was listed as a new food ingredient by the National Health Commission of China (National Health Commission of China, 2017). The recommended method of using ST dry leaves is brewing with a suggested dosage of up to 10 g/day based on dry product. Until now, published preclinical safety assessments on ST and its extract are still rare, and the understanding of the potential toxicity of ST is still limited. A long-term toxicity study reported that blood glucose level in Wistar rats were significantly decreased after treated with 2.0, 1.0, and 0.5 g/kg water-extract of ST leaves for 13 and 25 weeks. The AST and ALT activities were significantly increased in rats treated with 2.0 g/kg ST extract for 25 weeks but reduced to a level comparable to the control group 2 weeks after discontinuing administration. Hepatic cell edema was observed in rats treated with 2.0 g/kg ST extract for 25 weeks but cells edema was not observed 2 weeks after discontinuing administration. These results indicate that long-term treatment with ST extract could lead to reversible damage to the liver function of rats (Zeng et al., 2010). Other studies have reported little or no cytotoxicity of ST extract (Wang et al., 2020; Lin et al., 2023). The current study evaluated the toxicological potential of ST extract in a 90-day repeated dose subchronic oral toxicity study in Sprague-Dawley rats.

During the 90-day treatment period, no treatment-related general clinical observations were recorded. The body weights and food intake of rats in all treatment groups were comparable to those of the control group, indicating that the ST extract did not have a significant effect on the weight or appetite of rats during the experiment.

Several statistically significant fluctuations in blood hematological and serum biochemical indexes were observed in the subchronic toxicity study. However, the levels of these measurements remained within historical ranges in our laboratory and were consistent with reported values for SD rats. Therefore, these changes were not deemed treatment-related or of toxicological concern. A previous toxicity study reported decreased blood glucose levels in Wistar rats after being treated with ST extract for 13 and 25 weeks (Zeng et al., 2010); this effect was not observed in the 90-day toxicity study described in this paper. Possible explanations for this discrepancy include the use of different strains and varying methods for extracting ST leaves. Previous studies have reported that various extraction methods yield extracts with different components (Liu HY. et al., 2021; Liu Y. et al., 2021).

For urinalysis, a few positive values, including WBC, PRO, BLD, Cr, Ca, and MA, were recorded in rats from both the treatment and control groups. The absolute and relative weights of the kidneys were comparable across all groups. In the meantime, mild cell infiltration in the renal cortex observed in the kidneys of the treatment groups was similar to that of the control. These results indicate that the ST extract had minimal nephrotoxicity in rats.

In the gross examination, no apparent pathological lesions were observed in rats from all groups, and no specific tissues or organs were identified. In the histopathological examinations, inflammatory cell infiltration was observed in portal tract areas, along with fatty degeneration of hepatocytes in rats treated with 2000 mg/kg of ST extract. These changes were considered to be spontaneous lesions, as the occurrences of these minor histopathological changes were relatively low (≤30%) and comparable to those of the control group, and no treatment-related abnormalities in liver function indices were observed. Other than liver and kidney, no histopathological lesions were observed, which is consistent with a previous study that reported treatment with ST extract up to 2 g/kg for 25 weeks did not induce treatment-related histopathological changes in rats (Zeng et al., 2010).

## 5 Conclusion

In conclusion, the results of the 90-days subchronic oral toxicity study support the safety for the repeated oral consumption of the water extract of “Sweet Tea,” *L*. *litseifolius*. The no-observed-adverse-effect level of ST extract was considered to be 2,000 mg/kg/day for both male and female SD rats.

## Data Availability

The data presented in the study are deposited in the Figshare repository, accession: https://doi.org/10.6084/m9.figshare.25965574.
